# Adult Inflammatory Multi-System Syndrome Mimicking Kawasaki Disease in a Patient With COVID-19

**DOI:** 10.7759/cureus.11750

**Published:** 2020-11-28

**Authors:** Boniface Malangu, Javier A Quintero, Eugenio M Capitle

**Affiliations:** 1 Internal Medicine, Rutgers New Jersey Medical School, Newark, USA; 2 Rheumatology, Rutgers New Jersey Medical School, Newark, USA

**Keywords:** kawasaki disease (kd), pediatric inflammatory multisystem syndrome, sars-cov-2 and covid-19

## Abstract

We describe a 46-year-old male with severe acute respiratory syndrome coronavirus 2 (SARS-CoV-2) infection who presented as a Kawasaki-like syndrome with features including prolonged fever, bilateral conjunctivitis, oral mucosal swelling, diffuse erythematous rash, cervical and hilar lymphadenopathy, as well as cardiovascular complications and multi-organ failure. There are several reports of a similar clinical entity mimicking Kawasaki disease (KD) in the pediatric population, which has been termed Pediatric Inflammatory Multisystem Syndrome Temporally Associated with SARS-CoV-2 (PIMS-TS) by the Royal College of Pediatric and Child Health. To our knowledge, to date, there has been only one case report of COVID-19 presenting as KD in an adult patient.

## Introduction

Coronavirus disease 2019 (COVID-19) was officially declared a pandemic on 11 March 2020 [1]. Since the emergence of the first cases in China in late 2019, there have been more than six million cases and over 370 thousand deaths worldwide, as of 31 March, 2020 [2-3]. The COVID-19 pandemic is a rapidly evolving global healthcare crisis, and the disease can present with a wide range of signs and symptoms. Patients can be asymptomatic carriers or present with severe illness requiring intensive medical management [4-5]. Even though it mainly manifests as viral pneumonia, COVID-19 can present with multiple extrapulmonary and multi-systemic involvement, including cardiovascular, gastrointestinal, hepatobiliary, renal, hematologic, cutaneous, and neurological complications [6-11]. There are also multiple reports about unusual manifestations of SAR-CoV-2 infection, including a new clinical entity termed: Pediatric Inflammatory Multisystem Syndrome Temporally Associated with Severe acute respiratory syndrome coronavirus 2 (SARS-CoV-2) (PIMS-TS), a Kawasaki-like disease in children that has been reported in countries such as Italy and the UK [12-14]. Here, we present a case of an adult male with SAR-CoV-2 infection that presented with signs and symptoms mimicking Kawasaki disease (KD).

## Case presentation

A 46-year-old Latinx man with no significant past medical history presented with two months of persistent shortness of breath associated with subjective fever, sore throat, dry cough, general malaise, and recent onset pleuritic chest pain. The patient also complained of one month of diffuse nonpruritic skin rash, mostly involving his back, abdomen, pelvis, and bilateral upper extremities. He mentioned a recent trip to his homeland of the Dominican Republic three months prior and works at a convenience store, but he denied any known sick contacts. He also denied: high-risk sexual activity, illicit drug use, weight loss, hemoptysis, orthopnea, paroxysmal nocturnal dyspnea, or lower extremity edema.

Approximately six weeks prior to this presentation, the patient completed an empiric antibiotic course for suspected community-acquired pneumonia after presenting to the ER with a mild cough, shortness of breath, and diarrhea. At that time, the patient was febrile and chest X-ray showed bilateral interstitial infiltrates. He was otherwise stable and was discharged home with antibiotics.

**Figure 1:**
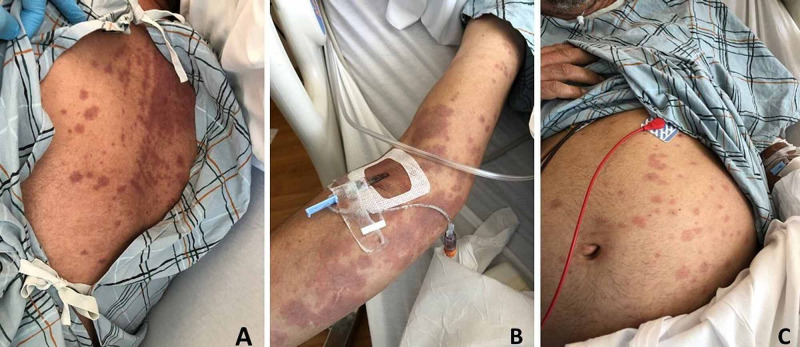
Coalescing macular rash on the back (A), right arm (B), and abdomen (C). The photos were taken on the fourth hospital day.

On this presentation, the patient was found to be febrile with a temperature of 39.1°C. He was also tachycardic with heart rate fluctuating between 130 and 180 beats per minute and mild hypoxia with pulse oximetry of 91% while on room air; otherwise, he remained normotensive. On exam, the patient was awake and in no acute distress. He was noted to have bilateral conjunctival injection without exudates and mild oral mucositis. Neck exam was remarkable for mild bilateral cervical lymphadenopathy, but no jugular venous distention or apparent thyromegaly. Cardiovascular exam revealed tachycardia with an irregularly irregular rhythm without significant murmur or peripheral edema. Lungs were clear to auscultation. Skin exam revealed a coalescing erythematous macular rash, predominantly scattered around upper extremities, abdomen, back, and groin (Figure 1). A rash with perioral distribution was also noted on the face. No joint tenderness and swelling were noted. Abdominal exam was without organomegaly. Neurology exam was unremarkable. 

**Figure 2:**
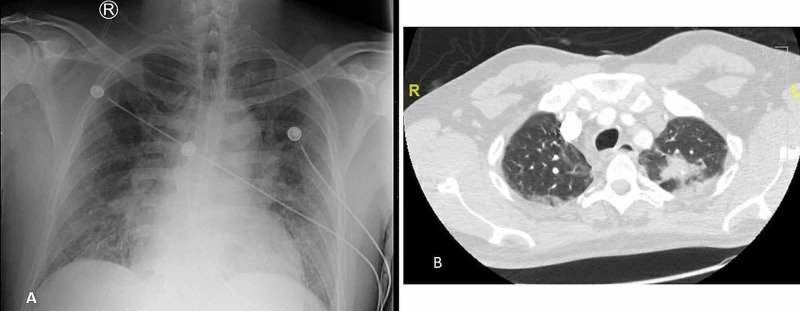
Chest X-ray, AP view showing a hazy right middle lobe opacity and bibasilar linear opacities (A). CT angiography revealing patchy consolidations on bilateral lung apices; more prominent on the left side than the right (B). AP, anteroposterior

**Figure 3:**
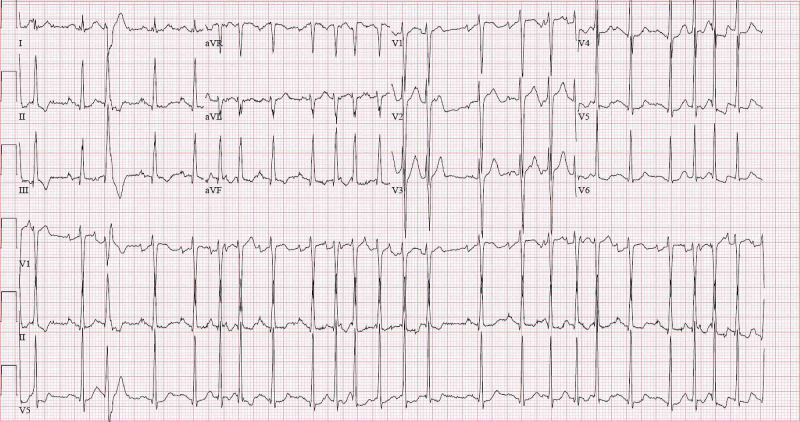
A 12-lead electrocardiogram obtained on presentation showing atrial fibrillation with rapid ventricular response. EKG, electrocardiogram

Laboratory results revealed white blood cell (WBC) count of 10.3 x 103/μL, hemoglobin 16.5 g/dL, thrombocytopenia of 119 x 103/μL, D-dimer of 4400 ng/mL, lactate dehydrogenase (LDH) of 253 u/L, C-reactive protein (CRP) of 74 mg/L, and ferritin of 827 ng/mL. Other abnormal laboratory findings included activated partial thromboplastin time (aPTT) 61 s, fibrinogen 664 mg/dL, creatinine 1.6 mg/dL, creatine kinase 773 u/L, alanine aminotransferase, and aspartate transaminase 76 U/L and 57 U/L, respectively. Urinalysis was remarkable for pyuria (WBC 10/HPF), moderate hematuria, proteinuria (protein 30 mg/dL), and negative for both nitrite and leukocyte esterase. On imaging studies, chest X-ray revealed a middle lobe opacity and basilar linear opacities, and CT angiography showed patchy consolidations on bilateral lung apices (Figure 2). Electrocardiogram (EKG) showed atrial fibrillation with rapid ventricular response (RVR) (Figure 3). Transthoracic echocardiogram showed left ventricular eccentric hypertrophy with reduced ejection fraction of 31%. Subsequent cardiac MRI revealed bilateral perihilar lymph nodes, but no suspicion for infiltrative disorders like sarcoidosis or amyloidosis.

Extensive infectious workup, which included specific testing for HIV, streptococcus, legionella, tuberculosis, syphilis, pneumocystis, histoplasma, cryptococcus, aspergillus, malaria, babesia, and strongyloides, was unrevealing. Blood and sputum cultures, viral hepatitis panel, respiratory panel, as well as acid-fast bacilli smear with culture were also negative. SARS CoV-2 rapid polymerase chain reaction (PCR) testing was negative on two occasions, but the patient eventually tested positive for SARS CoV-2 IgG antibodies. He underwent bronchoscopy on day six of hospitalization. Bronchial washings were also unremarkable; cytology was negative for malignant cells. Workup to evaluate for underlying autoimmune conditions included testing for antinuclear antibodies, anti-neutrophil cytoplasmic antibodies, complements C3 and C4 levels, rheumatoid factor, anti-glomerular basement membrane antibody, and cryoglobulins were also unremarkable. Skin biopsy of the rash showed interface dermatitis with negative antibody or complement deposition on immunofluorescence.

The patient remained febrile for at least the first six hospital days consecutively. His health continued to decline with progressive renal insufficiency, liver injury, and thrombocytopenia but remained hemodynamically stable throughout the hospital stay. The trend in inflammatory markers continued to increase during the first six days as well. He received supportive care and completed a 10-day course of empiric treatment for community-acquired pneumonia and sepsis with cefepime combined with doxycycline. For atrial fibrillation, anticoagulation therapy was initiated with apixaban. On the eighth hospital day, the patient began to show signs of clinical improvement as evidenced by the resolution of his initial symptoms and fever. From this day forward, the thrombocytopenia, renal, and liver function continued to improve until full recovery. On day 10, the patient actually converted back to sinus rhythm as he continued his recovery. On hospital day 12, he was discharged home in stable condition with close outpatient follow-up.

## Discussion

Kawasaki disease, also known as mucocutaneous lymph node syndrome, is a medium-vessel vasculitis that predominantly affects children less than five years old. This syndrome is rarely seen in adults. As of 2010, there have been only 80 reported cases of typical KD in adults [15]. Diagnostic criteria for KD, originally postulated by Tomisaku Kawasaki in 1967, requires the presence of fever lasting ≥ 5 days plus at least four out of the following five clinical findings: bilateral bulbar conjunctival injection, cervical lymphadenopathy >1.5 cm diameter, polymorphous rash, oral mucosa changes (such as injected or fissured lips, pharyngeal erythema, or strawberry tongue), and peripheral extremity changes (such as palmar and plantar erythema or edema). Although not part of the original clinical criteria, cardiovascular complications like coronary artery involvement, cardiac arrhythmias, and ventricular dysfunction are well-known complications of KD [16].

The patient described here presented with a Kawasaki-like syndrome with a prolonged fever of greater than five days in duration along with oral mucosal swelling, bilateral nonpurulent conjunctivitis, a diffuse rash, mild bilateral cervical lymphadenopathy, and evidence of hilar lymphadenopathy. He also had signs of cardiovascular involvement, manifested as new-onset atrial fibrillation, and left ventricular dysfunction. Similar to the recently described PIMS-TS or Kawasaki-like disease associated with COVID-19 in children who tested negative for SARS-CoV-2 PCR but have SARS-CoV-2-positive antibodies, this constellation of findings, along with evidence of unregulated inflammatory response and multi-organ failure seen weeks after a viral prodrome, raises the suspicion of a new clinical entity of COVID-19 mimicking KD [8-10, 17-18]. Our patient’s initial infectious workup was unrevealing, including negative SARS-CoV-2 PCR testing. However, he had serological evidence of COVID-19 infection. As our understanding of SARS-CoV-2 continues to evolve, this case shows us that COVID-19 may also masquerade as KD in the adult patient.

## Conclusions

Adult inflammatory multi-system syndrome in SARS-Cov-2 infection is an emerging disease state and one that warrants more research especially since the pandemic is not abating. Treatment for KD primarily involves the uses of aspirin and intravenous immune globulin (IVIG). Our patient did not require escalated treatment as he eventually recovered with general supportive care and close monitoring. In refractory cases of KD, immunosuppression with high doses of systemic glucocorticoids or even cyclosporine can be considered. The role of these agents for adult patients with COVID-19 mimicking KD remains to be determined. Furthermore, the role of steroids, particularly dexamethasone, which has been shown to be an effective treatment for COVID-19, could also be investigated as a potential treatment for adult patients with COVID-19 mimicking KD.
